# Sybil Attack-Resistant Blockchain-Based Proof-of-Location Mechanism with Privacy Protection in VANET

**DOI:** 10.3390/s24248140

**Published:** 2024-12-20

**Authors:** Narayan Khatri, Sihyung Lee, Seung Yeob Nam

**Affiliations:** 1 Department of Information and Communication Engineering, Yeungnam University, Gyeongsan 38541, Republic of Korea; narayankhatrig@ynu.ac.kr; 2 School of Computer Science and Engineering, Kyungpook National University, Daegu 41566, Republic of Korea; sihyunglee@knu.ac.kr

**Keywords:** VANET, proof-of-location (PoL), sybil attack, privacy, smart contract, blockchain

## Abstract

In this paper, we propose a Proof-of-Location (PoL)-based location verification scheme for mitigating Sybil attacks in vehicular ad hoc networks (VANETs). For this purpose, we employ smart contracts for storing the location information of the vehicles. This smart contract is maintained by Road Side Units (RSUs) and acts as a ground truth for verifying the position information of the neighboring vehicles. To avoid the storage of fake location information inside the smart contract, vehicles need to solve unique computational puzzles generated by the neighboring RSUs in a limited time frame whenever they need to report their location information. Assuming a vehicle has a single Central Processing Unit (CPU) and parallel processing is not allowed, it can solve a single computational puzzle in a given time period. With this approach, the vehicles with multiple fake identities are prevented from solving multiple puzzles at a time. In this way, we can mitigate a Sybil attack and avoid the storage of fake location information in a smart contract table. Furthermore, the RSUs maintain a dedicated blockchain for storing the location information of neighboring vehicles. They take part in mining for the purpose of storing the smart contract table in the blockchain. This scheme guarantees the privacy of the vehicles, which is achieved with the help of a PoL privacy preservation mechanism. The verifier can verify the locations of the vehicles without revealing their privacy. Experimental results show that the proposed mechanism is effective in mitigating Sybil attacks in VANET. According to the experiment results, our proposed scheme provides a lower fake location registration probability, i.e., lower than 10%, compared to other existing approaches.

## 1. Introduction

Today, vehicular ad hoc networks (VANETs) are an important component of intelligent transportation systems (ITS). VANETs are a subclass of mobile ad hoc network (MANET), which provides communication opportunities between the vehicles or between the vehicles and the roadside infrastructure. The VANET communication provides a data-sharing facility to maintain the security and reliability of the transportation system. It provides a wide range of services, including an entertainment facility, driving assistance, collision avoidance, traffic management, and other safety applications. Nowadays, modern vehicles provide enhanced sensing and networking functionalities with larger computation and storage options [1]. VANET utilizes these facilities to perform its operations. VANETs support a variety of communication modes, including vehicle-to-vehicle (V2V), vehicle-to-infrastructure (V2I), vehicle-to-network (V2N), vehicle-to-pedestrian (V2P), and vehicle-to-grid (V2G). The wireless technology used for this communication can be IEEE 802.11p [2] dedicated short range communication (DSRC) or cellular networks (i.e., 4G, 5G) [3,4]. On-board units (OBUs) facilitate message exchange between vehicles using V2V mode, whilst vehicles and infrastructures communicate using V2I communication technology. There are two types of messages in VANETs: basic safety messages and event messages [5]. Basic safety messages contain a vehicle’s velocity, time, location, elevation, heading, acceleration, yaw rate, steering wheel angle, length, width, and other information, which are periodically broadcast to neighboring vehicles. The event messages comprise road event information, such as traffic congestion or accidents, which is transmitted over long ranges using V2I technology [6]. These messages can be an important source for potential safety applications and traffic management for the traffic control authority [7]. The dissemination of false messages can mislead the vehicles and will be a risk to the safety of passengers or other road users. Thus, it is important to evaluate the accuracy of messages transmitted by a vehicle and infrastructure in VANETs. Furthermore, the rise in connected and autonomous vehicles (CAEVs) poses security threats as smart vehicles connect through the internet. Hence, the security of VANETs is crucial, and the related issues need to be dealt with carefully.

This paper proposes a proof-of-location (PoL) mechanism for mitigating Sybil attacks in VANETs. A Sybil attack is a form of attack in which a malicious node generates multiple fake identities called Sybil nodes to influence the functioning of a network. In a Sybil attack, malicious vehicles create multiple fake identities that can generate messages resembling the ones from valid IDs. In vehicular ad hoc networks, attackers can disrupt the functioning of the network by generating fake messages. Consequently, the whole transportation system will be unreliable, and the safety of road users cannot be guaranteed. Furthermore, the generation of multiple pseudonyms by a single malicious driver and their location information can pose the risk of a Sybil attack in VANET [7]. Thus, we propose a blockchain-based PoL scheme for ensuring safety and security in VANET. We will store the legitimate vehicle identity and location information inside the blockchain smart contract. Blockchain is a secured and decentralized ledger that cannot be easily compromised due to its design structure. The information inside the blockchain is stored in the form of blocks and each block is linked with each other using the cryptographic hashes forming the chain structure. A block comprises the hash of the previous block, timestamp, nonce, transaction data, etc. In our approach, the transaction data will contain the location proofs of the vehicles passing the polling process. The transactions recorded inside the blockchain is irreversible, i.e., in order to change one block of record, all other subsequent block records need to be changed. Thus, blockchain is a secured and decentralized database that can guarantee the security of information stored inside it.

Various studies have been conducted on the detection of Sybil attacks in VANET. Most of these studies reconstruct vehicle trajectories based on message exchanges between a vehicle and the infrastructure [7,8,9]. If two or more trajectories are not sufficiently distinct, the corresponding trajectories are assumed to be formed for the same Sybil node. Although these studies can accurately identify a Sybil attack, they can impose an extra burden on the infrastructure, especially in dense traffic with frequent reports. Additionally, the way these studies handle message exchanges can reveal users’ real identities and their location information, raising privacy concerns. Thus, we propose a Sybil attack mitigation mechanism for VANET, which is both lightweight and privacy-preserving. In the proposed PoL mechanism, we store valid vehicle location information inside the smart contract. This smart contract, which is maintained by road side units (RSUs), act as the ground truth for the location verification of the vehicles. We design a computational puzzle-based polling mechanism in which each vehicle needs to solve a puzzle before submitting their location information to nearby RSUs. The RSU issues a unique puzzle to the vehicles residing in its communication zone. If the hash of the computation puzzle submitted by each vehicle is less than the difficulty target, RSU stores the vehicle’s location information into the smart contract. The details of the computation puzzle design and the difficulty target will be discussed in a later section. The key idea for addressing the Sybil attack problem in VANET is that a vehicle can solve a single puzzle at a time, assuming that each vehicle has a single CPU assigned for the puzzle computation task. Today, single-core ECUs are still dominant for VANET communication as compared to multi-core ECUs [10]. According to [10], most vehicle functions, such as heating, ventilation and air conditioning (HVAC), or powertrain control, are developed for single-core ECUs in model-based design languages. We will deal with the Sybil attack detection in multi-core ECU environments in our future work. Furthermore, the identity and location information of vehicles inside the smart contract is encrypted with a PoL privacy preservation mechanism, thereby preserving the privacy of vehicles.

The main contributions of this paper can be summarized as follows:

- A new proof-of-location (PoL) mechanism is proposed for verification of vehicle location in VANET and the prevention of Sybil attacks. It does not impose an extra burden on the trusted authorities to manage the polling process as required in other works.
- The privacy of vehicles, including identity and location, is important. Thus, we handle the privacy issues with the help of a PoL privacy preservation mechanism. With the help of this approach, the trajectory of a given vehicle *V* cannot be traced by a third party *T* easily. And the validity of the location of a given vehicle *V* can be proved to a third party.
- Experiments and simulation show that the proposed PoL approach can mitigate Sybil attack problems in VANETs. Furthermore, the use of smart contracts will ensure the privacy of the vehicles and other entities involved in the VANET system.

The abbreviations used in this paper are provided in the Abbreviations Section. The rest of the paper is organized as follows. Section 2 investigates related work. Section 3 provides the design goals, and the system framework is given in Section 4. Section 5 provides a detailed explanation of the proposed system. Section 6 provides the experimentation and performance evaluation. Section 7 and Section 8 provide security analysis and conclusion, respectively.

## 2. Related Work

Various studies have been conducted on the detection of Sybil attacks in vehicle networks. One group of studies rebuilds vehicle trajectories and examines if any pair of trajectories shares similarities (i.e., if the trajectories belong to the same Sybil node).

Sybil attack detection deploying proofs of work and location has been studied by Baza et al. [7]. RSUs provide a signed time-stamped tag as proof of a vehicle’s location. Each vehicle needs to solve a PoW puzzle to obtain a location tag when it approaches the next RSU. In this way, after a vehicle passes *n* numbers of RSUs, a trajectory is formed, which acts as the vehicle’s anonymous identity. Then for detecting Sybil attack, two main heuristics are developed based on traverse time limit and trajectory length limit to decide whether two trajectories are distinct or agree with each other.

Benadla et al. [8] suggested a method for detecting Sybil attacks in vehicular fog networks utilizing RSSI and blockchain. The Sybil attack is detected at two levels: first, by estimating the distance between the fog node and the vehicle OBU using the RSSI signal intensity. A Sybil node is identified when a request comes from two vehicles with a distance difference less than the threshold, which is the smallest distance that can separate two vehicles from each other. Second, the position tag provided by fog nodes determines the trajectory of vehicles. The possibility of a Sybil attack is determined when two trajectories exist in the fog nodes at the same time.

Chang et al. [9] proposed a Sybil attack detection mechanism for vehicular networks called Footprint. In this method, RSUs provide anonymous messages as proof of a vehicle’s presence at a specific time. The trajectory of a vehicle’s anonymous identification is determined by messages collected from the encountered RSUs. However, this approach is vulnerable to RSU compromise attack as it is infrastructure dependent, i.e., RSU [7]. Hackers can create multiple fake trajectories by exploiting this vulnerability in areas with sparse RSU presence.

Although these studies [7,8,9] can effectively discover Sybil reports, correlating different pairs of reports and rebuilding trajectories impose an excessive burden on the infrastructure, especially in the case of dense traffic scenarios. In contrast, our proposed work prevents Sybil attacks rather than detecting them afterward. We mandate puzzle-solving before sending a valid location, thereby limiting each vehicle to sending a single valid report at a time. Moreover, this work focuses on maintaining the privacy of a vehicle’s location information inside the smart contract table of the blockchain-based VANET system as compared to the previous studies.

The blockchain-based privacy preservation mechanism in the VANET environment has been studied in the literature [11,12,13,14,15,16].

In [11], RSUs maintain the relationship between a vehicle’s pseudonym and real identity on the blockchain. The information included within the blockchain is public. And there is no mechanism for privacy protection of the vehicle, and thus the vehicles’ privacy might be compromised. The certificate authority (CA) stores the pseudo ID of each vehicle corresponding to the real identity in its database. Thus, this is a centralized approach and has a risk of single point of failure.

Alharthi et al. [12] proposed a biometrics-based blockchain solution, BBC, for the privacy preservation and security of VANET. The biometrics information, such as a fingerprint, is used to verify the integrity of the message and verify the identity of the message sender anonymously. This biometrics-based authenticated certificate generation for vehicles can be a viable solution for maintaining security and privacy in the VANET system. However, this study does not address how we can preserve the location privacy of the vehicles inside the blockchain.

It is very important to preserve the location privacy and user’s identity information in VANET, as emphasized by Li et al. [13]. In this paper, the authors design a blockchain-based identity and location privacy protection mechanism for VANET. The basic safety messages are used for communication among vehicles and infrastructures in a VANET system. They designed a dynamic threshold encryption mechanism for identity privacy protection and k-anonymity unity for location privacy preservation of those safety messages while uploading data to the VANET blockchain. This scheme is not feasible for vehicular networks because of high processing power requirements and communication overhead as a result of its complex design structure.

Qureshi et al. [14] developed a blockchain-based, privacy-preserving authentication mechanism for intelligent transportation systems. The proposed system includes smart contract-based data storage and access control mechanisms to ensure the privacy and security of ITS users. Furthermore, access control policies are utilized to direct the on-demand function to supply the necessary services.

Ahmed et al. [15] proposed a blockchain-based event message authentication and trust management system for VANETs. The authentication of messages guarantees that only legitimate vehicles take part in the communication process. However, this work does not consider the security attack mitigation strategy in VANETs as compared to our proposed work.

Location privacy preserving system using blockchain technology has been proposed in [16]. They use vehicle certificates to request location information from the RSU. The use of a certificate will preserve the privacy of vehicle information. This blockchain scheme may be vulnerable to sophisticated attacks from hackers. Also the privacy of the information can be leaked if we store information in the public blockchain.

Yang et al. [17] proposed a blockchain-based trust management model for VANETs. The joint proof-of-work and proof-of-stake consensus mechanism has been developed for finding the nonce for the hash function (PoW). The RSUs will take part in maintaining this blockchain, which stores the reputation value for each vehicle involved in the VANET event message communication. However, generating ratings for the vehicles involved in communication is cumbersome, as discussed in [18].

Javaid et al. [19] proposed a smart contract-based secured framework for trust management in the internet of vehicles environment. They also proposed the identity privacy preservation mechanism for vehicles by the application of physically unclonable functions (PUFs). A PUF is the hardware fingerprint that uniquely identifies the vehicles in the proposed system. The drawback with this approach is that the hardware fingerprint is vulnerable to theft or vandalism thereby raising other security and privacy concerns.

The blockchain-based solutions [14,15,16,17,19] proposed the trust management and privacy preservation mechanism for VANETs. However, these works do not deal with the security attacks (i.e., Sybil attack) mitigation strategy. The proposed PoL scheme is a hybrid blockchain-based solution that deals with both privacy preservation and security attack mitigation for VANETs. Thus, our work is a unique solution as compared to the previous works.

Maffiola et al. [20] proposed a simulation tool called GOLIATH, which is used for real-time data collection for intelligent transportation systems (ITSs). The proposed blockchain-enabled data collection mechanism allows vehicles to share their and their neighbors’ positions, which will be recorded on the ledger in a distributed fashion. This helps to mitigate the single point of failure of the centralized system for trust and data management in the ITS systems. This is the only simulator tool for VANET traffic data collection and management with the functionality of attack scenario simulation. Thus, we have selected this simulator for the simulation of our proposed work.

Most of the existing proof-of-location approaches have been dealing with either Sybil attack mitigation or privacy protection separately. In addition, these approaches usually did not consider a highly sophisticated Sybil attack case, where multiple legitimate vehicle IDs are available to the attackers via hacking. Furthermore, earlier approaches suggest solutions that are computationally expensive or impose extra burden on the transportation management authority. Thus, this study attempts to fill this gap by considering the Sybil attack mitigation and privacy protection together.

## 3. Requirements for Proof-of-Location Systems

This section discusses the requirements that proof-of-location systems need to satisfy in terms of privacy, authenticity, and Sybil attack mitigation.

### 3.1. Anonymous Identity and Privacy

The proposed system should be able to maintain the vehicle’s anonymous identity through the generation of pseudoIDs (PIDs). These PIDs are used by the vehicle each time it reports the event information to the RSUs. The privacy of the driver and the vehicle’s location is an essential issue that needs to be resolved while deploying blockchain-based solutions in the VANET environment. The vehicle identities, such as plate number and VIN number, are private and are maintained through the application of a PoL-based privacy preservation mechanism.

### 3.2. Authenticity

Each entity in the proposed framework will be authenticated at response of the handshaking procedure. Vehicles, RSUs, and verifiers will be authenticated on request based on the identity information stored in the DMV.

### 3.3. Sybil Attack

A Sybil attack is a dangerous attack that will hamper the smooth operation of an intelligent transportation system. The attack scenario assumes that the hacker has access to multiple private keys, which they can use for generating fake vehicle IDs. These identities can be acquired through a variety of techniques, including obtaining unused vehicle IDs from the used car market, breaking into the vehicle registrar’s system, and hacking into the other user’s vehicular systems. The proposed PoL system, which includes a puzzle computation-based polling mechanism, will help to mitigate Sybil attacks in VANETs.

## 4. Participating Entities of the System

**Figure 1:**
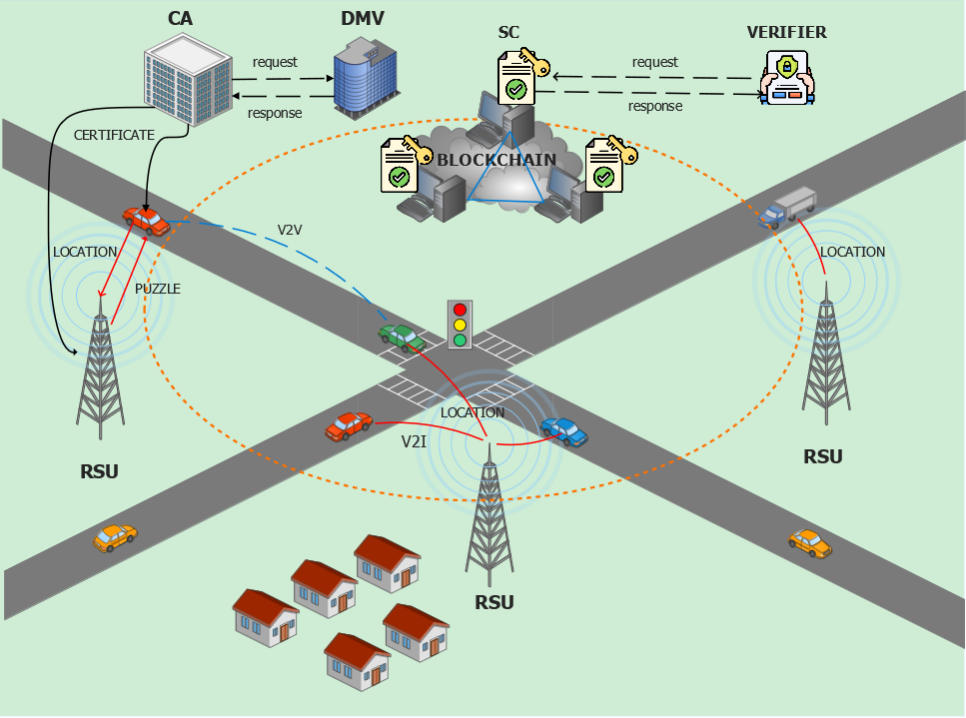
The system framework of the proposed work.

This paper proposes a Proof-of-Location scheme that mitigates Sybil attacks in VANETs. The system comprises various entities, which work together for the efficient management of traffic event information and location verification purposes. The system architecture of the proposed scheme is shown in Figure 1.

### 4.1. Vehicles

Vehicles are the primary components of VANET message exchange. They are involved in the transmission of safety and event messages to other vehicles and road infrastructures such as RSUs. Vehicles tackle a unique challenge each time they pass the communication zone of RSUs to verify their identity in the VANET system. Furthermore, they are involved in the creation of anonymous identities, which can be utilized to protect the privacy of the vehicles involved in event message generation. In our proposed approach, vehicles within the communication range of neighboring RSUs communicate location data as shown in Figure 1. RSUs further store this information in the smart contract. Authorities such as the police department or insurance companies can be verifiers who utilize this information to resolve disputes in the event of an emergency or a traffic accident.

### 4.2. Road Side Units (RSUs)

RSUs are road infrastructures that have communication capabilities and can take part in location verification for vehicles. They communicate with the vehicle OBUs using the DSRC protocol or cellular networks [18,21]. In contrast to vehicles, they have high storage capacity and processing power. RSUs generate puzzles for the vehicles and verify if the puzzle solution is correct or not. If a vehicle solves the puzzle, then the event information containing location data is stored in a smart contract table by RSUs. We assume that the RSUs are deployed with a similar distance between the consecutive ones along the road section and it cannot be easily compromised by hackers. These RSUs maintain a dedicated blockchain to store the information about the passing vehicle in the table of the smart contract.

### 4.3. Certificate Authority (CA)

A certificate authority is a trusted authority that provides a certificate for the vehicles and RSUs in the VANET system [22]. They can authenticate the real identity of the vehicle and RSUs with reference to the DMV. As part of the initial stage, the CA participates in vehicle and RSU registration as well as authentication in collaboration with the DMV.

### 4.4. Department of Motor Vehicles (DMV)

The DMV is a crucial part of the VANET framework. It is responsible for storing the vehicle’s true identity, such as the vehicle identification number (VIN) or plate number, the manufactured date, the buyer’s name, and so on, and delivering it to trusted authorities such as CA whenever necessary [12]. Similarly, we assume that the DMV stores the true identity of RSUs in its database.

### 4.5. Smart Contract (SC)

A smart contract is a piece of code that is invoked by RSUs and stores the location information of vehicles [19]. RSUs can write to the SC table, but vehicles and other entities can only read the information in it. The information inside the smart contract table cannot be stored in plain text since it can leak the privacy of vehicles. As a result, this information is hashed or transformed to make it difficult to infer the vehicle information using a privacy preservation mechanism.

### 4.6. Prover

A prover is an entity that wants to prove its location information to the verifier [23]. The vehicles in the VANET system acts as prover.

### 4.7. Verifier

The location information of the vehicle can be verified by a third party, such as a police department or insurance companies [23]. They need location information of vehicles in case of accidents or to track malicious vehicles on the road. The validity of the event message from a vehicle can be inferred by proving its location at a specific time. Thus, this information might be critical for authorities to manage the transportation system reliably.

### 4.8. Transactions

The transactions involved in our proposed work comprise a vehicle’s identity information, position information, and road event information [4]. The transactions consist of timestamps and are signed by the initiator’s private key. To avoid third parties inferring the trajectory of the vehicles, the information is hashed and secured with a PoL-based privacy preservation mechanism.

### 4.9. Blockchain

The blockchain is a secured, tamper-resistant distributed ledger maintained by RSUs for storing the smart contract tables. The blockchain consists of growing blocks of data that are linked together through cryptographic hashes [24,25]. In this paper, we store the position information of vehicles in the smart contract table of blockchain. The information inside the smart contract table is in encrypted form and cannot be easily revealed. However, the location information of the vehicle at a specific time can be proved to the third-party verifier. The block containing vehicle location information is stored in the blockchain based on the consensus of the majority of RSUs in the blockchain network.

## 5. The Proposed Proof-of-Location Mechanism

**Figure 2:**
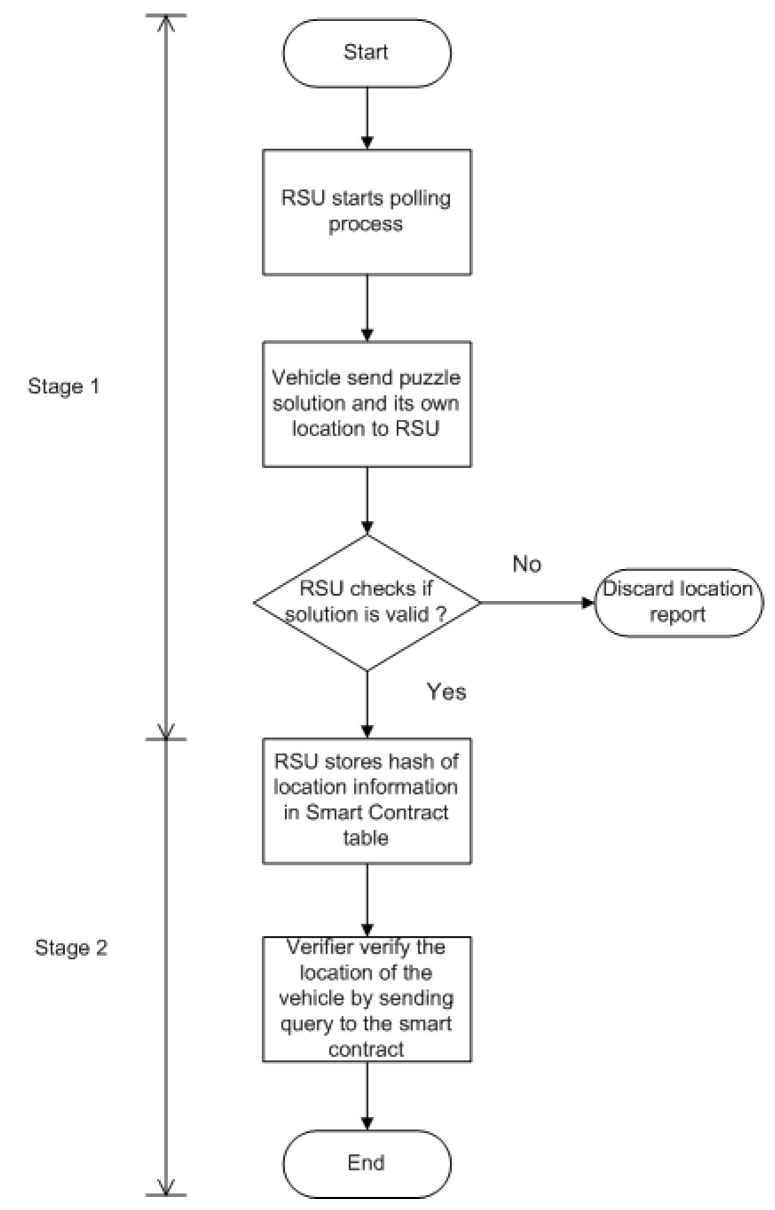
Flowchart describing the outline of the proposed PoL scheme.

Figure 2 provides the flowchart illustrating the principle of the proposed proof-of-location mechanism. In Stage 1, RSU starts the polling process, and the vehicle that solves the computational puzzle within the time limit has a chance to register its own location with the help of RSU. This computational puzzle lowers the possibility of a Sybil attack. In Stage 2, the vehicle that provided the correct solution to the computational puzzle will store its own location information inside the smart contract of the blockchain without exposing its own identity. The verifier can verify the location of a specific vehicle while preserving the privacy of the vehicle. A pseudo-code illustrating the functioning of the proposed work is given in Algorithm 1. The detailed explanation of the proposed mechanism is provided in Section 5.3 and Section 5.4 with list of notations in Table 1.
**Algorithm 1** Algorithm for the proposed PoL mechanism1:Input: location report ($L_{i}$)2:Output: proof-of-location (PoL) for $V_{i}$3:**Initialization:** list of Vehicles ($V_{i}=\left[\right]$), list of RSUs ($R_{j}=\left[\right]$), Global−PID=[], puzzle solution ($N_{i}=\left[\right]$), smart contract ($SC_{R_{j}}$), verifier ($X_{i}$), *g*, *q*4:**Stage 1:**5:$RSU(R_{j})$ starts the polling process for vehicles in its communication zone6:**for** each $V_{i}$**in** list $R_{j}$ **do**7:    RSU−$R_{j}$ issues random puzzle to $V_{i}$8:    Vehicle−$V_{i}$ solve the puzzle and sends solution $N_{i}$ with $L_{i}$ to $R_{j}$9:    **if** $N_{i}$ is valid and on time **then**10:        $R_{j}$ stores location $L_{i}$ in $SC_{R_{j}}$11:    **else if** $N_{i}$ is invalid and not in time **then**12:        $R_{j}$ discard location $L_{i}$13:    **end if**14:**end forend if**15:**Stage 2:**16:$V_{i}$ sends Global−PID to verifier $X_{i}$ through a secured channel17:**for** each Global−PID received **in** list Global−PID[] **do**18:    Verifier−$X_{i}$ query time $t_{i}$ and location $L_{i}$ to $SC_{R_{j}}$19:    $X_{i}$ computes the validity check function $f(t_{i},L_{i},d)$ and verifies the equality of $Z_{i}=f\left(t_{i},L_{i},d\right)$20:    **if** equality is valid, i.e., the location $L_{i}$ sent by vehicle matches with the information in $SC_{R_{j}}$ **then**21:        The location $L_{i}$ of $V_{i}$ is verified22:    **else if** equality is not valid, i.e., the location $L_{i}$ sent by vehicle do not match with the information in $SC_{R_{j}}$ **then**23:        The location $L_{i}$ of $V_{i}$ is not verified24:    **end if**25:**end for**

### 5.1. System Initialization

The proposed system consists of five entities, vehicle ($V_{i}$), RSU ($R_{j}$), smart contract ($SC_{R_{j}}$), verifier ($X_{i}$), and certificate authority (CA). $V_{i}$ represents the *i*th vehicle (i=1,2,……,n), and $R_{j}$ represents the *j*th RSU (j=1,2,……,m). The $SC_{R_{j}}$ at *j*th RSU is used for storing location information of vehicles traversing its communication zone. The CA generates certificates for vehicles and RSUs, which are signed by its private key. We assume that CA generates certificates and keys for both vehicles and RSUs based on the elliptic-curve cryptography (ECC) scheme of public-key cryptography. The notations used in the proposed system are shown in Table 1.

### 5.2. Vehicle and RSU Registration

At first, vehicles and RSUs need to be registered to the CA in order to obtain the certificate. The detailed steps are explained in the following steps.

*Step 1: Vehicle* Vehicle $V_{i}$ sends vehicle identification number $VIN_{i}$ along with other credentials to the CA for the first time registration process. The CA verifies the real identity of the vehicle in collaboration with the Department of Motor Vehicles (DMV). If the credentials of the vehicle $V_{i}$ are verified, the CA provides a certificate to the vehicle sending the request as in (1).

(1) $$H_{0}=\left(Cert_{V_{i}}\right)$$

*Step 2: RSU* Similarly RSU sends its identity information i.e., $RID_{j}$ and other information to the CA for registration. The CA verifies the identity of the RSU from the DMV and generates the certificate $Cert_{R_{j}}$ to the RSU as in (2).

(2) $$H_{1}=\left(Cert_{R_{j}}\right)$$

### 5.3. Computational Puzzle-Based Polling Mechanism

**Figure 3:**
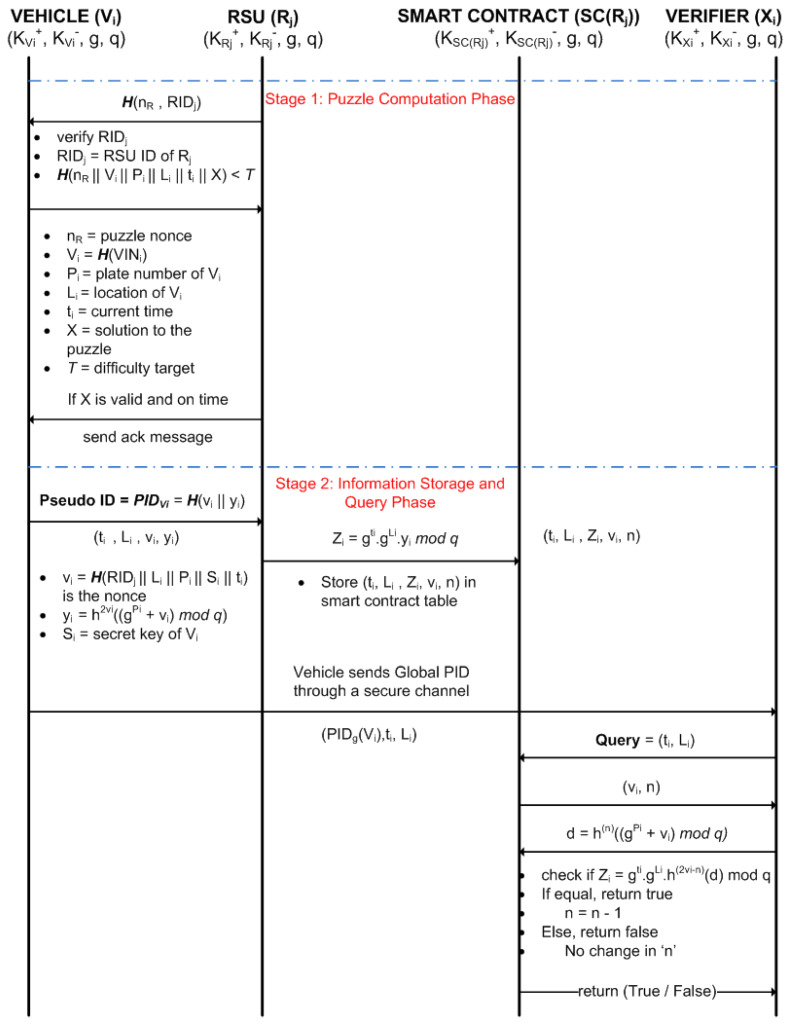
Message exchanges between vehicle, RSU, smart contract, and verifier.

The message exchange among the entities of the proposed scheme is shown in Figure 3. The first stage is the puzzle computation phase. In this phase, RSU generates a puzzle with a nonce ($n_{R}$) and $RID_{j}$ and sends it to the vehicles entering its communication zone. This message is signed with the private key of the RSU, $R_{j}$ i.e., $K_{R_{j}}^{-}$ and consists of its certificate $Cert_{R_{j}}$. The vehicle $V_{i}$ verifies the message using the public key of $R_{j}$, i.e., $K_{R_{j}}^{+}$ incorporated in the certificate. The vehicle confirms the message’s authenticity since the RSU signing the message with its private key can only be decoded using its public key. Then, the vehicle solves the puzzle and returns the solution to the RSU along with the nonce $n_{R}$ signed with its private key $K_{V_{i}}^{-}$. $R_{j}$ verifies the message using the public key of the vehicle $V_{i}$, i.e., $K_{V_{i}}^{+}$. If RSU can verify the message and the nonce returned is the same as the one sent before in the handshaking process, it authenticates the vehicle. Then, the RSU extracts the puzzle solution *X* and checks if it meets the difficulty target *T*. If the criteria are satisfied, the vehicle is given permission for further message exchange process in Stage 2.

The difficulty should be adjusted in such a way that the average puzzle computation time does not exceed the threshold. The details of the polling mechanism and the average puzzle computation time for each vehicle are provided in Section 5.5. A solution to the puzzle means an answer that meets the criteria for the difficulty target. The lower the target value, i.e., a longer sequence of preceding zeros in the target, the more difficult the puzzle will be. Thus, the puzzle computation time and polling interval need to be determined carefully. The puzzle can be described as follows using (3).

(3) $$H(n_{R}||V_{i}||P_{i}||L_{i}||t_{i}||X)<T$$

where, *X* = solution to the puzzle, $n_{R}$ = puzzle nonce provided by the RSU $R_{j}$, $V_{i}$=$H(VIN_{i})$ = hash of the VIN of vehicle $V_{i}$, $L_{i}$ = location of $V_{i}$, $t_{i}$ = current time, $P_{i}$ = plate number of $V_{i}$, and *T* = difficulty target of the puzzle. The *H* function used for computing the puzzle can be the SHA-256 hash function. The puzzle computation process involves the vehicle sending its solution along with its location and time information. This will prevent the vehicles from precomputing the puzzle and using it for the PoL process. This makes our system more efficient for Sybil attack mitigation in VANETs.

### 5.4. Privacy-Preserving Proof of Location

In this section, we will explain in detail the privacy preservation mechanism for the proposed scheme. This is the second stage (i.e., information storage and query phase) of the proposed PoL scheme for message exchanges between the vehicle and other entities of the system, as shown in Figure 3. Initially, vehicles that solve the puzzle generate the Global PID for themselves, which is the global pseudo-identity of $V_{i}$ that will be utilized for its location verification by the verifier. The Global PID of $V_{i}$ is defined as follows:

(4) $$PID_{g}\left(V_{i}\right)=\left(g^{P_{i}}\;mod\;q\right)$$

where $P_{i}$ is the plate number of $V_{i}$. Then, the initiator vehicle will calculate two entities $v_{i}$ and $y_{i}$ for securely storing the location information inside the smart contract. $v_{i}$ is the nonce calculated as (5) and $y_{i}$ is calculated by hashing the summation of Global PID and $v_{i}$$\left(2v_{i}\right)$ times as (6). Here, the hash operation has been performed $2v_{i}$ times to ensure the privacy of the vehicle information. The application of hash operation multiple times helps us ensure the security of the proposed approach. We note that $L_{i}$ = location of vehicle $V_{i}$, $P_{i}$ = plate number of $V_{i}$, $S_{i}$ = secret key of $V_{i}$, $t_{i}$ = current time, $RID_{j}$ = ID of the RSU $R_{j}$. *g* is the generator and *q* is a selected prime number.

(5) $$v_{i}=H(RID_{j}\left|\right|L_{i}\left|\right|P_{i}\left|\right|S_{i}\left|\right|t_{i})$$

(6) $$y_{i}=h^{\left(2v_{i}\right)}\left(\left(g^{P_{i}}+v_{i}\right)mod\;q\right)$$

where $h^{\left(t\right)}\left(x\right)=h(h\left(\ldots \ldots h\left(x\right)\right)$ i.e., the hash operation has been performed *t* times. Then, the vehicle sends the tuple $\left(t_{i},L_{i},v_{i},y_{i}\right)$ to the correspondent RSU after signing it to store its location information. Then, the RSU will authenticate the sender of the message based on its public key. After that it will calculate $Z_{i}$ as (7) using a cryptographic hash function. However, the verifier can use it to verify that the PoL event information is produced from vehicle $V_{i}$. The RSU stores the tuple $\left(t_{i},L_{i},Z_{i},v_{i},n\right)$ in the smart contract table, which consists the PoL information for each of the vehicle that passed the polling process as shown in Table 2. *n* is a random number used for the purpose of one-time password during the handshaking procedure of the location verification system. The value of *n* will be randomly selected by the RSU, and the tuple $\left(t_{i},L_{i},Z_{i},v_{i},n\right)$ will be stored inside the table of blockchain by the RSU.


(7) $$\begin{array}{c} Z_{i}=g^{t_{i}}\;\cdot \;g^{L_{i}}\;\cdot \;y_{i}\;mod\;q\\ =g^{t_{i}}\;\cdot \;g^{L_{i}}\;\cdot \;h^{\left(2v_{i}\right)}\left(\left(g^{P_{i}}+v_{i}\right)\;mod\;q\right)mod\;q \end{array}$$

Let us consider that there is an accident on the road, and the vehicles involved in an accident need to prove their location to the verifier. The verifier acts as the mediator between the vehicle owner and authorities to solve conflicts, e.g., to determine if the insurance claim made by a vehicle involved in an accident is legitimate or not. The vehicles involved in conflicts can provide false location data to hide their wrongdoing. Thus, location verification of the vehicle is important. In order to prove its location, the vehicle will send its Global PID to the verifier along with its time and location information. We assume that the Global PID will be sent through the secure channel, e.g., by encrypting with the public key of the receiver. Then, the verifier $X_{i}$ can query the smart contract with a request message consisting of the tuple $\left(t_{i},L_{i}\right)$ for location verification of vehicle $V_{i}$ at some time $t_{i}$. The verifiers can be organizations such as police departments or insurance companies, which have the right to know about the location information of the vehicles involved in incidents. If there is a request from these authorities, the smart contract will respond with the nonce $v_{i}$ generated by $V_{i}$ at time $t_{i}$ along with the random number *n* generated by the RSU to the related authorities. The verifier will calculate *d* as (8) using Global PID, $v_{i}$, and *n* obtained from the vehicle and the smart contract. Then, the verifier will send *d* to the smart contract for validation of its knowledge of the existence of $V_{i}$ with a certain Global PID at a specific time. The smart contract will process the value of *d* and returns true if the equality in (9) is valid.

(8) $$d=h^{\left(n\right)}\left(\left(g^{P_{i}}+v_{i}\right)mod\;q\right)$$

(9) $$Z_{i}=g^{t_{i}}\;\cdot \;g^{L_{i}}\;\cdot \;h^{\left(2v_{i}-n\right)}\left(d\right)mod\;q$$

The equality of (9) can be shown in the following way. If we put *d* in (8) into *d* in the right side of (9), then the right side of (9) can be expressed as follows:

$$\begin{array}{c} (g^{t_{i}}\;\cdot \;g^{L_{i}}\;\cdot \;h^{\left(2v_{i}-n\right)}\left(d\right))mod\;q\\ =g^{t_{i}}\;\cdot \;g^{L_{i}}\;\cdot \;h^{\left(2v_{i}-n\right)}\left(h^{\left(n\right)}\left(\left(g^{P_{i}}+v_{i}\right)mod\;q\right)\right)mod\;q\\ =g^{t_{i}}\;\cdot \;g^{L_{i}}\;\cdot \;h^{\left(2v_{i}\right)}\left(\left(g^{P_{i}}+v_{i}\right)mod\;q\right)mod\;q\\ =Z_{i} \end{array}$$

Thus, we have shown that the equality of (9) is valid only when the appropriate value of *d* is provided by the verifier, and this value of *d* can be obtained only when the verifier knows the Global PID of $V_{i}$. Thus, (9) will prove two things. Firstly, it proves the location information of the specific vehicle with a given Global PID to the verifier at some RSU. Secondly, it illustrates whether the verifier’s knowledge of that specific vehicle’s information is correct or not. We will update *n* by decrementing the value by 1 (i.e., n=n−1) if the location of a particular vehicle has been verified by the verifier. This is necessary to prevent a replay attack on the last step. For example, if *n* is incremented by 1, then the next *d* can be easily derived by hashing the previous *d* one more time because of the following:

$$\begin{array}{c} d_{new}=h^{\left(n+1\right)}\left(\left(g^{P_{i}}+v_{i}\right)mod\;q\right)\\ =h(h^{\left(n\right)}\left(\left(g^{P_{i}}+v_{i}\right)mod\;q\right)\\ =h(d_{old}) \end{array}$$

Let us consider a case a verifier wants to verify the location of a specific vehicle to solve a criminal case for instance. In this practical scenario, the vehicles will provide their Global PID, time, and location information to the verifier. Then, the verifier will query the information against the smart contract table to determine the validity of the information provided. The verifier cannot easily infer the information of the specific vehicle based on the information contents on the smart contract table or the users information. This is because of the use of hash functions to derive the value of $Z_{i}$. The third party cannot easily reverse the value of $P_{i}$ or $L_{i}$. Furthermore, finding the value of $P_{i}$ given *g*, *q*, and $y_{i}$ from the provided Global PID is hard because it is a discrete logarithm problem [26]. Therefore, the proposed PoL-based privacy preservation mechanism can prove the location of a specific vehicle at a given RSU without revealing its real identity.

### 5.5. Analysis of Polling Interval and Puzzle Computation Time

**Figure 4:**
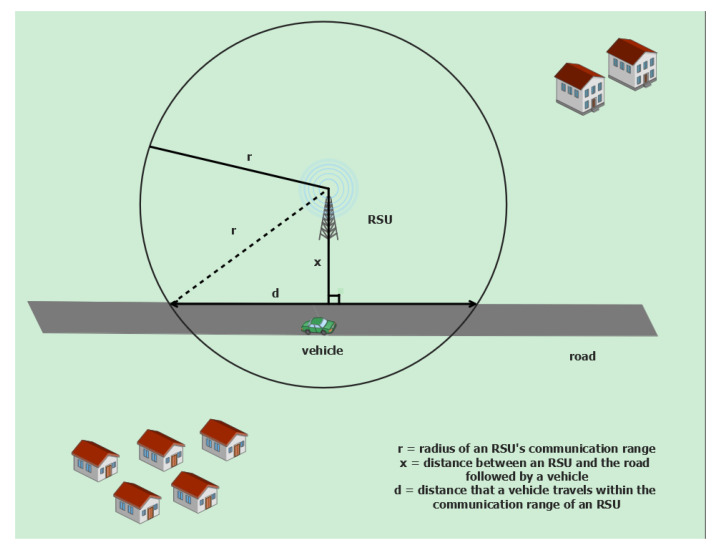
Distance traversed by a vehicle when the communication range of the RSU and the distance between the RSU and the road is given.

In this subsection, we analyze the polling interval and puzzle computation time. Figure 4 shows the distance traced by a vehicle while the vehicle moves within the range of a given RSU. *r* is the radius of the communication range of the given RSU. *d* is a random variable denoting the distance that a vehicle travels within the communication range of the RSU. *x* is a random variable denoting the distance between the RSU and the road followed by the given vehicle. To simplify the analysis, we will assume that each road is straight without a curve in this paper. If *x* is larger than *d*, then there is no interaction between the corresponding RSU and the given vehicle, and thus, we need not consider those RSUs in this analysis. Thus, we will assume that *x* is uniformly distributed between 0 and *r*, i.e.,

(10) $$f_{x}\left(x\right)=\left\{\begin{array}{cc} \frac{1}{r}, & 0\leq x\leq r,\\ 0, & otherwise. \end{array}\right.$$

From Figure 4, we can easily derive the following relation between *d* and *x*:

(11) $$d=2\sqrt{r^{2}-x^{2}}$$

From (10) and (11), we can obtain

$E\left[d\right]=E\left[E\left[d|x\right]\right]=\int_{0}^{r}E\left[d|x=x\right]f_{x}\left(x\right)dx$,

(12) $$E\left[d\right]=\int_{0}^{r}2\sqrt{r^{2}-x^{2}}\times \frac{1}{r}dx.$$

If we put $\frac{x}{r}=\sin\theta$ and apply the technique of change of variable to the above integration, then we have the following:

(13) $$E\left[d\right]=2r\int_{0}^{\frac{\pi }{2}}\cos^{2}\theta d\theta =\frac{\pi }{2}r$$

If the average vehicle speed is $v_{a}$, then the average sojourn time of the vehicle ($t_{a}$) can be calculated as follows:

(14) $$t_{a}=\frac{E[d]}{v_{a}}=\frac{\pi r}{2v_{a}}$$

under the straight road assumption within the range of the given RSU. If we want to sample this vehicle at least once before it leaves the communication range of the RSU, the polling interval needs to be shorter than $t_{a}$ in (14). If we decide to sample each vehicle about two times considering the margin, then the polling interval $T_{po}$ can be calculated as

(15) $$T_{po}=\frac{t_{a}}{2}=\frac{\pi r}{4v_{a}}$$

The power consumption of the ECUs involved in the polling process can be controlled by the complexity of the computational puzzle. If we want to maintain the utilization of ECUs involved in the polling process lower than α , then the puzzle complexity needs to be adjusted such that the average puzzle computation time ($T_{pu}$) satisfies the following constraint:

(16) $$T_{pu}\leq \alpha T_{po}=\frac{\pi \alpha r}{4v_{a}}$$

We can consider an example scenario for the computation of polling interval and average puzzle computation time as follows. Let us consider the following parameters:

(17) r=500m

The average vehicle speed is assumed to be

(18) $$v_{a}=40\;\mathrm{km}/h=\frac{40*10^{3}\;m}{60*60\;s}=11.1\;m/s.$$

Then, the polling interval will be calculated as follows,

(19) $$T_{po}=\frac{1}{2}\times t_{a}=\frac{\pi \times 500}{4\times 11.1}=35.4\;s.$$

If α=0.05, i.e., only 5% of ECUs power is utilized, then we have

(20) $$T_{pu}\leq \alpha \times T_{po}=0.05\times 35.4=1.77\;s.$$

Thus, the average puzzle computation time $T_{pu}$ can be adjusted based on the value of α. If the value of α is high, the puzzle computation time increases. Thus, adjusting the value of α for puzzle computation will help to mitigate the Sybil attack in VANET.

From now on, let us discuss the relation between the complexity of the computational puzzle and the puzzle computation time ($T_{pu}$). The computational puzzle can be represented as follows:

(21) h(π||x)<t

where π represents the values determined by the given condition, *t* is the threshold, and *x* is the only variable. *X* is a random variable denoting the number of attempts required until the first success in the computational puzzle. Let us assume that h(.) is an ideal uniform random hash function to simplify the analysis, and the hash value is *l* bits long. If $t=2^{l_{1}}$, then the probability that a value of *x* satisfies (21) is $p=\frac{2^{l_{1}}}{2^{l}}=2^{l_{1}-l}$ , and we can easily know that *X* has the following geometric distribution,

(22) $$Pr\left(X=k\right)=\left\{\begin{array}{cc} {\left(1-p\right)}^{k-1}p, & k\geq 1,\\ 0, & otherwise. \end{array}\right.$$

In addition, the expectation of *X* is well-known as

(23) $$E\left[X\right]=\frac{1}{p}=2^{l-l_{1}}$$

When δ represents a time required to run one hash function and one comparison in (21), let us think about the probability that the total puzzle computation time (δX) is less than a given threshold $T_{pu}$ in (16). The following relation can be obtained as follows.

(24) $$Pr\left(\delta X\leq T_{pu}\right)=Pr\left(X\leq \frac{T_{pu}}{\delta }\right)=\sum_{k=1}^{T_{pu}/\delta }{\left(1-p\right)}^{k-1}p$$

or,

(25) $$Pr\left(\delta X\leq T_{pu}\right)=1-{\left(1-p\right)}^{\frac{T_{pu}}{\delta }}$$

If we want to keep this probability higher than or equal to $p_{t}$, then we have

(26) $$1-{\left(1-2^{l_{1}-l}\right)}^{\frac{T_{pu}}{\delta }}\geq p_{t}$$

because $p=2^{l_{1}-l}$. If all the parameters, *l*, δ, $T_{pu}$, and $p_{t}$, are fixed, the parameter $l_{1}$ which controls the complexity of the puzzle can be determined by solving the inequality (26), and we can obtain

(27) $${\left(1-2^{l_{1}-l}\right)}^{\frac{T_{pu}}{\delta }}\leq 1-p_{t}$$

or

(28) $$1-2^{l_{1}-l}\leq {\left(1-p_{t}\right)}^{\frac{\delta }{T_{pu}}}$$

Therefore,

(29) $$l_{1}\geq l+\log_{2}\left(1-{\left(1-p_{t}\right)}^{\frac{\delta }{T_{pu}}}\right)$$

If the value of $p_{t}$ is large up to 1, then $l_{1}$ increases up to *l* according to the above relation, and this means that the computation puzzle gets very easy and the whole system can be vulnerable to the Sybil attack again. Thus, the control parameter $p_{t}$ needs to be determined appropriately while avoiding large values close to 1. In the above relation, the value of δ can be different depending on the vehicles or processors on them, and thus, this relation may not be applied to practical situations directly. However, this relation can be useful to find an initial value on a specific machine, which can be updated adaptively as in Bitcoin case.

## 6. Experimentation and Performance Evaluation

### 6.1. Smart Contract Implementation and Gas Cost Analysis

To evaluate our scheme, we developed smart contract for vehicle and RSU registration, update of vehicle information, and query of the information by the verifier. We have tested our smart contract using the Sepolia test network of the Ethereum blockchain. Sepolia is the default testnet for smart contract-based blockchain application development [27]. We used remix IDE for the purpose of debugging and executing our smart contract. For fascilitating the use of Ethereum blockchain, we have added MetaMask chrome extension in our browser [27]. MetaMask is an Ethereum wallet that holds an Ethereum account and is used for transactions between accounts. Furthermore, it allows us to view transaction details on Etherscan.

**Figure 5:**
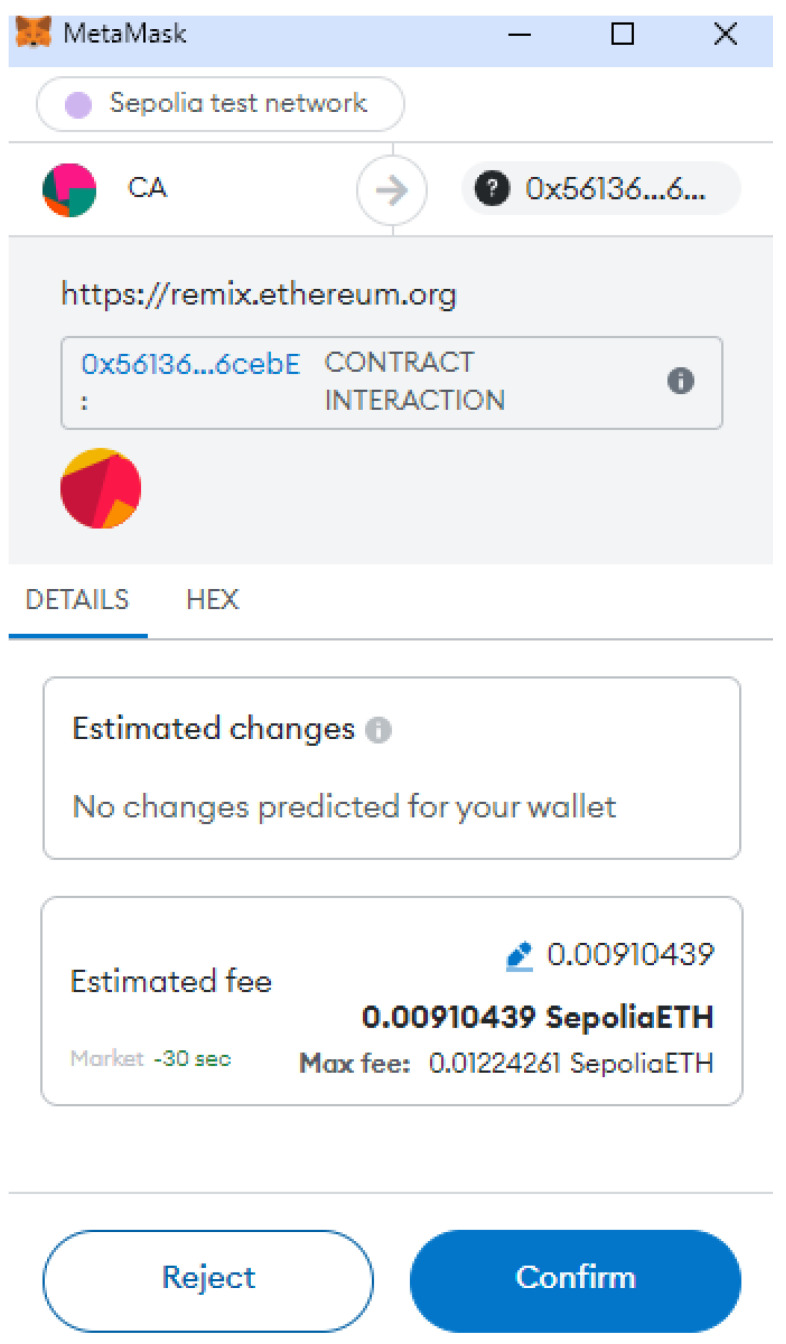
Vehicle registration in the Ethereum test network.

**Figure 6:**
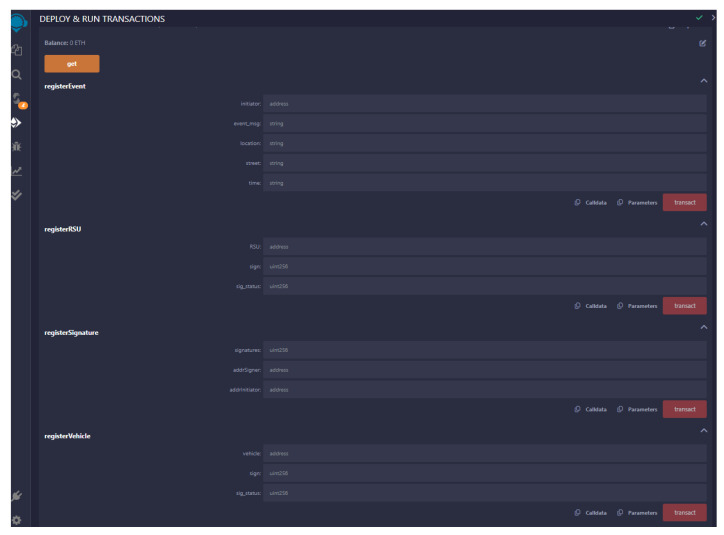
Remix IDE environment for VANET blockchain.

**Figure 7:**
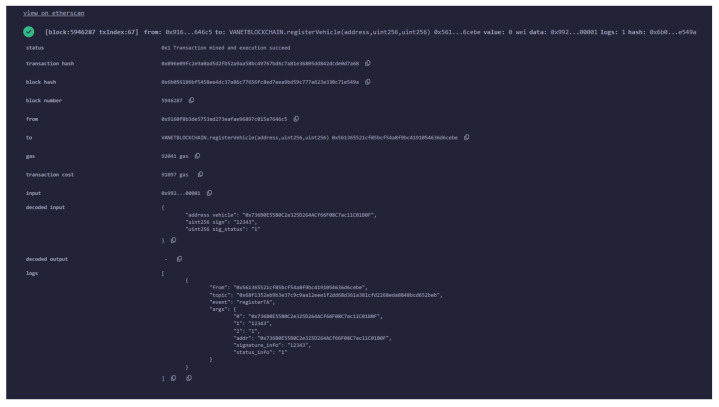
Execution logs for vehicle registration.

**Figure 8:**
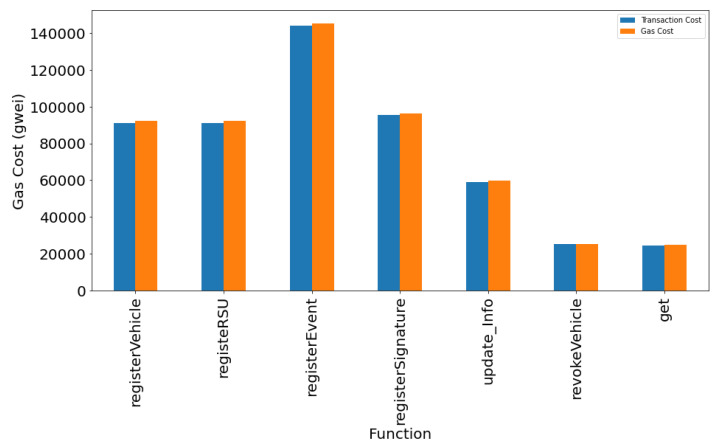
Transaction cost and Gas cost for smart contract functions.

In the beginning, we generate three accounts for CA, RSU, and Vehicle1 in MetaMask. We have implemented various functions such as registerVehicle(), registerRSU(), getVehicleList(), getRSUList(), registerEvent(), viewEvent(), registerSignature(), revokeVehicle(), update_Info(), and get() for simulation of our work in the Ethereum blockchain. These functions are invoked from the particular accounts for demonstration of the VANET blockchain environment. The functions registerVehicle() and registerRSU() are used for the registration of vehicles and RSU, respectively. This operation is performed through the help of a trusted authority. We can obtain the list of vehicles and RSUs registered in the blockchain using getVehicleList() and getRSUList() functions, respectively. The function registerEvent() facilitates the initiator vehicle to register an event to the nearby RSU. Similarly, viewEvent() will help users to retrieve the event information initiated by a particular vehicle. The function registerSignature() will register the signature of the vehicles, sending the event information along with the address of the signer. Some misbehaving vehicles can be revoked by CA using the revokeVehicle() method. RSUs can also update the vehicle information in smart contracts using update_Info() function. The verifier can verify and obtain the required information from the smart contract using the get() method. Figure 5 shows the MetaMask popup for confirmation of the contract deployment when the function registerVehicle() was invoked. If we confirm the estimated fee, the transaction will be mined in the Ethereum blockchain. The smart contract has been written in solidity programming language using the remix IDE environment. Figure 6 shows the snippet of the front end to interact with the Ethereum blockchain with the smart contract code. Figure 7 provide the transaction logs that are generated as a result of registerVehicle function execution in the console. Figure 8 shows the transaction and gas cost for various operations performed in Ethereum blockchain.

We now analyze the computation cost of the proposed smart contract for the VANET blockchain implementation simulation environment in the Sepolia test network of Ethereum. Table 3 provides the gas cost of the functions that we implemented for the VANET blockchain smart contract. The list includes Gas Consumed, Ether Cost (in ETH), and the actual cost in USD. We can see that the operations performed consume less amount of gas and the cost in USD is minimal. Thus, the proposed system can be feasible for maintaining the blockchain-based vehicular network environment.

### 6.2. Simulation Results

#### 6.2.1. Simulation Setup

**Figure 9:**
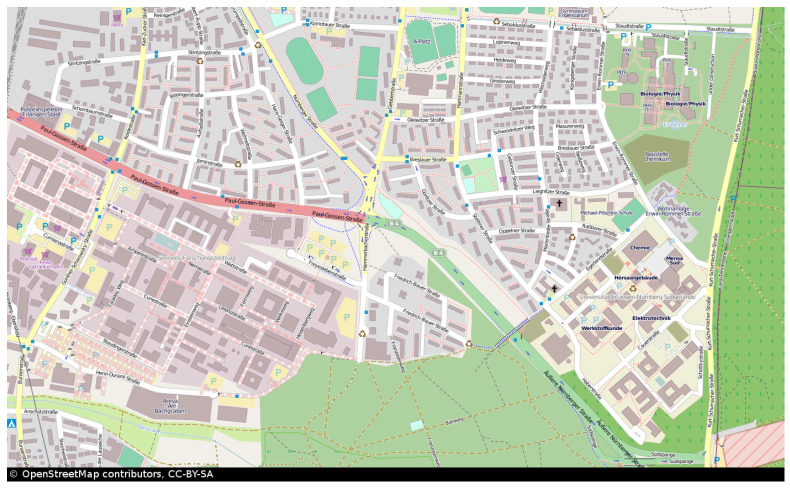
Map of Erlangen used for VANET traffic generation.

The proposed blockchain-based PoL scheme has been evaluated through simulation using Veins, Eclipse SUMO (Simulation of Urban Mobility), and OMNeT++. Veins 5.2 is an open source software for vehicular ad hoc network simulations which is based on SUMO and OMNeT++ library [28]. It uses IEEE 802.11p and IEEE 1609.4 [29] DSRC/WAVE protocol at the network level. SUMO provides real-time traffic scenarios involving traffic lights, buses, trucks, pedestrians, and trains, as well as other road infrastructures such as road side units (RSUs) [30]. It is free software for generating VANET traffic scenarios, which can be further utilized for network simulations. OMNeT++ is a C++-based simulation framework for performing network simulations [31]. We have used the map of erlangen for performing the VANET simulation as shown in Figure 9 [32]. The simulation consists of 100 vehicles interacting with each other with a maximum speed of 40 km/h. The simulation parameters for our simulation work are shown in Table 4. The blocksize is 50,000 bytes and the block generation time is set to 15 s in our simulation.

#### 6.2.2. Evaluation Metrics

The metrics used for our evaluation are as follows.

- ***Successful Sampling Probability*** is the probability that a vehicle is sampled by an RSU at least once while it passes the communication range of the RSU. It is computed as follows.
  (30) $$P\left(S\right)=\frac{\#\;\mathrm{of}\;\mathrm{vehicles}\;\mathrm{solving}\;\mathrm{the}\;\mathrm{puzzle}}{\#\;\mathrm{of}\;\mathrm{vehicles}\;\mathrm{within}\;a\;\mathrm{given}\mathrm{RSU}}$$
- ***Fake Location Registration Probability (FLRP)*** is the percentage of fake locations accepted by a given RSU in a given time interval. It is computed as follows.
  (31) $$\begin{array}{c} P\left(FLRP\right)=\frac{\#\;\mathrm{of}\;\mathrm{fake}\;\mathrm{location}\;\mathrm{accepted}\;\mathrm{by}\;a\;\mathrm{given}\;\mathrm{RSU}}{\#\;\mathrm{of}\;\mathrm{total}\;\mathrm{location}\;\mathrm{accepted}\;\mathrm{by}\;a\;\mathrm{given}\;\mathrm{RSU}} \end{array}$$
- ***Malicious Block Insertion Probability*** is the ratio of malicious blocks mined over the total number of blocks mined in the proposed blockchain.
- ***Event Message Propagation Delay*** is the time required to authenticate an event message by a given RSU.

#### 6.2.3. Successful Sampling Probability vs. Polling Interval

**Figure 10:**
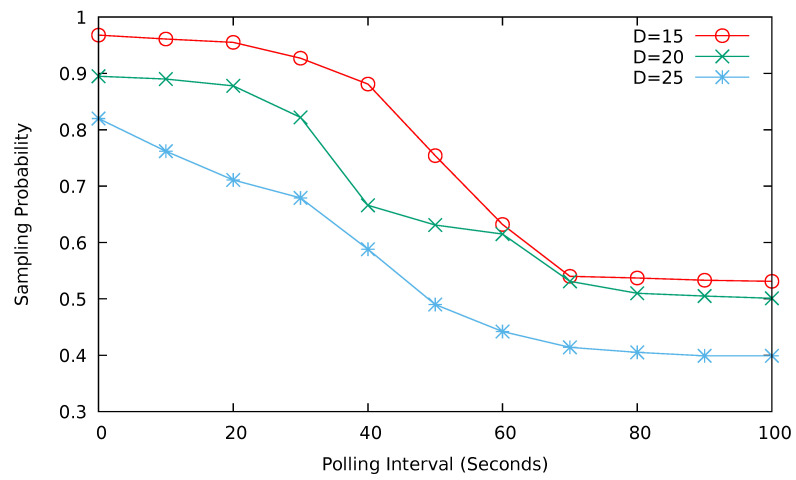
Sampling probability vs. polling interval (sampling time = 1 s, D = number of leading zeros of the difficulty target).

Figure 10 plots the graph showing the relation between sampling probability and polling interval based on the puzzle computation difficulty target *t*. In this simulation, we fix the sampling time, i.e., the poll response collection time, to 1 s. The difficulty target for the puzzle will be determined based on the hash algorithm used and the number of leading zeros in the hash prefix. Since the SHA-256 algorithm has 256 bits of the hash value, we have used $2^{256-D}$ as the target for the computational puzzle. *D* is the number of leading zeros for the target and it determines the puzzle difficulty. We can see from Figure 10 that as the value of *D* increases, the sampling probability decreases. This is due to the puzzle’s increased complexity, which causes vehicles to fail to solve the puzzle and report their location data. Since the target was $2^{l_{1}}$ in Section 5.5, we have $256-D=l_{1}$. We have shown the increase in $l_{1}$, which corresponds to the decrease in *D*, leads to the increase in the sampling probability. This analysis result agrees well with the trends in Figure 10.

When *D* is 15, i.e., the number of leading zeros is 15 and the polling interval is 30 s, the sampling probability is approximately 95%. In this case, the puzzle computation time will be less than 1 s. When the polling interval is short, the sampling probability is high. However, as the polling interval increases, the sampling probability decreases. Thus, we have selected 30 s as the polling interval for subsequent simulations.

#### 6.2.4. Successful Sampling Probability vs. Sampling Time

**Figure 11:**
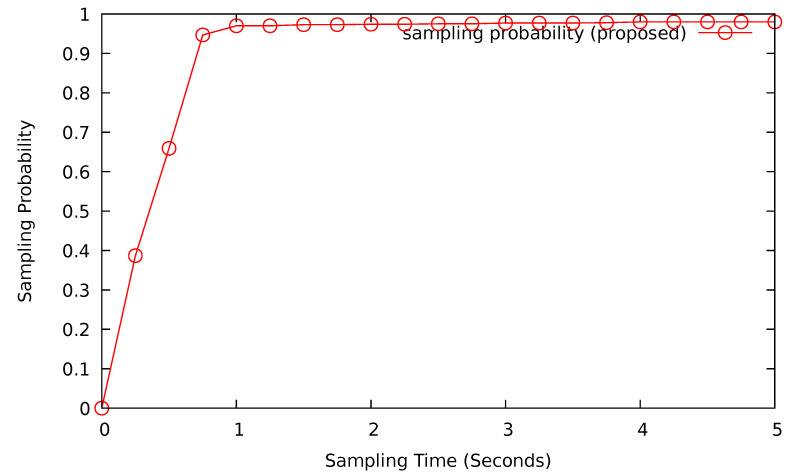
Sampling probability (polling interval = 30 s).

In this subsection, we will analyze the sampling probability by fixing the polling interval and difficulty of the network. The polling interval is fixed to 30 s, and the difficulty *D* will be 15 leading zeros. Figure 11 shows the sampling probability for various sampling times. When the sampling time is below 1 s, the probability tends to increase from a small number. As the sampling time increases over 1 s, the sampling probability remains consistent around 97%.

#### 6.2.5. Fake Location Registration Probability

**Figure 12:**
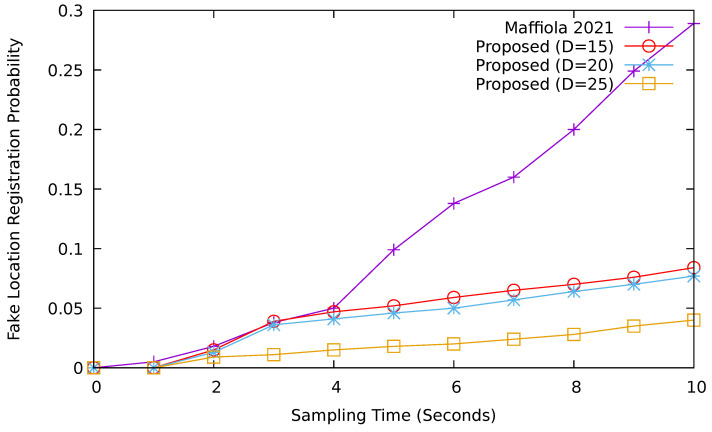
Fake location registration probability comparison (polling interval = 30 s) [20].

Figure 12 compares the fake location registration probability (FLRP) of our proposed scheme and GOLIATH for various sampling times. Here, we have fixed the polling interval to 30 s. The difficulty of the puzzle can affect the probability. The fake location registration probability is affected by two factors; sampling time and the difficulty of the puzzle. When the sampling time increases, the FLRP probability also increases. When the sampling time is higher, malicious vehicles can use it to solve a larger number of puzzles and register more fake location information. Thus, the probability goes on increasing. Furthermore, when the difficulty of the puzzle increases, the probability decreases as the vehicles cannot solve the puzzle easily. When the sampling time is around 1 s, the fake location registration probability is lower. Thus, we can say that the poll collection time of 1 s can be a reasonable choice for this case. And the polling interval of up to 30 s is acceptable for maintaining a higher sampling probability. If we limit the vehicles to utilize a limited amount of computational power (i.e., around 5%) to solve the puzzle within a limited sampling time period, we can mitigate the Sybil attack problem in VANET.

However, in the case of GOLIATH, the fake location registration probability shows a significant increase with the increase in the sampling time. There is no such polling mechanism in GOLIATH for avoiding a Sybil attack. Thus, the proposed approach performs better in terms of the fake location registration probability.

#### 6.2.6. Malicious Block Insertion Probability

**Figure 13:**
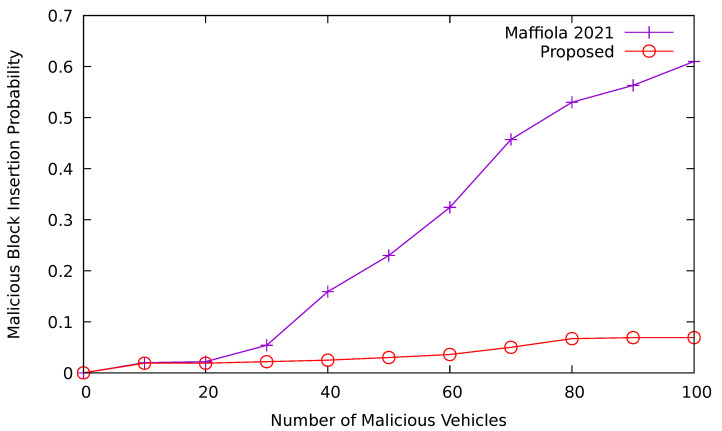
Malicious block insertion probability comparison [20].

Figure 13 compares the malicious block insertion probability of the proposed approach with that of GOLIATH [20]. In this simulation, we increase the number of malicious vehicles from 0 to 100 with the total number of vehicles fixed at 500 and observe the results. We can see from Figure 13 that the probability of malicious block insertion exhibits an increasing trend as the number of malicious vehicles increases in the case of GOLIATH. However, our proposed approach maintains this probability below 10%. In this simulation, we limit the vehicles to utilize 5% of their computational power to solve the PoL puzzle. As a result, the attacker cannot generate multiple solutions for the puzzle using fake location data to store false information inside the smart contract table. This is because the puzzle complexity is fixed, and vehicles can solve only one puzzle at a time with the given computational power and sampling time. Therefore, the malicious block insertion probability is maintained low with the proposed approach. Thus, the proposed approach performs better as compared to GOLIATH.

#### 6.2.7. Event Message Propagation Delay

**Figure 14:**
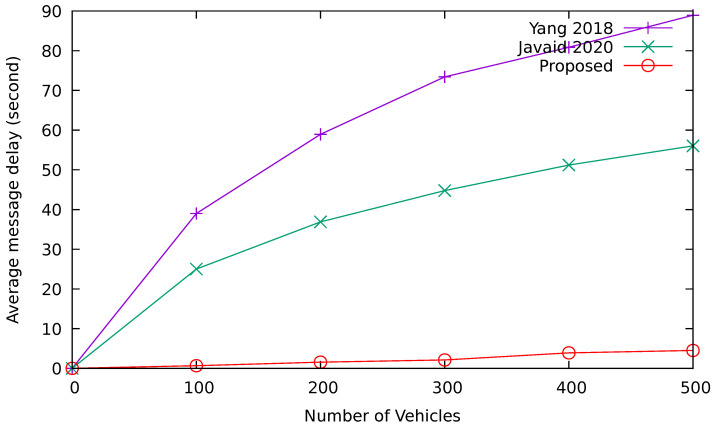
Event message propagation delay [17,19].

This is an important metric for the evaluation of the proposed VANET architecture. Delay is the time taken to deliver an event message from a vehicle to a neighboring RSU. Figure 14 compares the event message propagation delay among Yang et al. [17], Javaid et al. [19], and the proposed PoL scheme. We can see from the figure that the proposed approach maintains a lower delay as compared to other approaches despite the increase in the density of the vehicles. Therefore, the proposed approach meets the latency requirement for highly mobile VANET environments according to the simulation results. The low latency is achieved due to the computational puzzle-based polling mechanism. The vehicles try to solve the puzzle as soon as they enter the communication zone of an RSU. Thus, the delay is minimized.

## 7. Security Analysis and Discussion

### 7.1. Resistance to Sybil Attack

In this type of attack, malicious vehicles try to create many identities and generate false messages to misguide other vehicles or impose security threats on vehicles. The proposed approach in this paper mitigates this problem by developing a proof-of-location mechanism that makes vehicles solve a puzzle before reporting any event messages they observe on the road. The polling-based puzzle computation mechanism is designed for the vehicles entering the communication range of an RSU, which will induce vehicles to solve a single puzzle at a time. The polling interval and sampling time are fixed in order to sample more vehicles for the puzzle computation task, thereby reducing false location registration probability. This allows vehicles to utilize a small fraction of their computation power to solve the puzzle. A vehicle is restricted to solving a single puzzle at a given time with this approach. In this simulation, we have seen that the FLRP is almost zero when the polling interval is 30 s and the sampling time is 1 s. Furthermore, the sampling probability is higher up to 97% with the proposed approach. Thus, the proposed polling mechanism based on polling interval and sampling time with the given puzzle complexity helps to reduce the false location registration by malicious vehicles. Therefore, the proposed framework helps to mitigate the Sybil attack problem in VANETs.

### 7.2. Privacy Preservation

The proposed approach should ensure the privacy of a vehicle’s position, plate number, VIN number, and RSU information. For this purpose, we employ the PoL-based privacy preservation mechanism that guarantees the privacy of information storage in VANETs. The system design incorporates a unique pseudo ID generation mechanism that uses each vehicle’s secret key as well as other information each time it enters the RSU range. The pseudo ID generation procedure is random and unpredictable. These data are hashed in such a way that third parties cannot simply manipulate them. The use of the cryptographic hash function guarantees the privacy of the vehicles involved in the message communication. This is due to the fact that the one-way hash functions are irreversible, and reverse engineering, given the hash, requires a brute-force search. Furthermore, the verifier obtain Global PID through the secured channel and deriving plate number from it is a hard problem. Thus, the proposed PoL mechanism realizes the location verification of the vehicle while protecting its privacy.

### 7.3. Denial-of-Service (DoS) Attack

The proposed PoL scheme can withstand the denial-of-service attack launched by an attacker. The attacker might attempt to submit a large number of solutions to the puzzle in a short time interval. This problem can be resolved by employing a message signature and blacklist. When a vehicle submits its own answer for the puzzle it needs to be signed by its own private key. Thus, if a vehicle submits any incorrect solution to induce any computational burden on the proposed PoL system, then the vehicle will be registered in the blacklist. The blacklisted vehicle IDs and other information will be stored in the blockchain. Then, the attackers cannot easily launch the DoS attack because their vehicle IDs will be invalidated soon. It is not possible to send a signed message with an answer with a fake vehicle ID if the attacker does not know the private key of that vehicle. These countermeasures can help prevent from DoS attack in VANETs with the proposed approach. More details about the blacklist mechanism will be investigated in our future work.

### 7.4. Implementation and Scalability Issues

In this subsection, we will discuss scalability issues, computation overhead, and communication overhead associated with the proposed PoL mechanism. In this paper, we are not dealing with maintaining the trajectory of the vehicles. Managing the trajectories of vehicles can be costly and incur communication or computation overhead in the system, especially when the number of vehicles increases. In the proposed work, we use smart contracts to store the location information of vehicles instead of trajectory information. Our proposed scheme utilizes one of the existing frameworks, i.e., Ethereum, especially for the consensus mechanism. Thus, there is no additional computational cost to the blockchain system. In the proposed PoL mechanism, the computational cost for the polling process is limited because we are restricting the puzzle computation time for the vehicles. Furthermore, the polling process is not a continuous process because short polling times are separated by a long interval between them. Since the communication overhead happens only during polling times, and this polling time is maintained short in the proposed PoL scheme, the communication overhead is also limited. Thus, the proposed proof-of-location scheme can be scalable in a practical situation.

### 7.5. Discussion

Table 5 compares the proposed PoL scheme with the state-of-the-art literature in the field. We can observe from the table that the proposed work is effective for handling the Sybil attack and privacy problems in VANET communication as compared to the previous works.

## 8. Conclusions and Future Works

The security of VANET systems is critical and one of the most active research topics. Sybil attacks are considered to be one of the most serious types of attacks, which can pose security risks. This paper discusses the Sybil attack mitigation approach in VANETs. It is critical to take steps to reduce these types of dangers. Thus, this study proposes a computational puzzle-based polling strategy for mitigating the Sybil attack in VANETs. For this, vehicles need to solve a computational puzzle to report their location information to the nearest RSU. Experimental results demonstrate that the proposed PoL scheme can mitigate a Sybil attack in VANET. This is a novel approach with significant potential to safeguard the entire transportation system from attackers. The privacy of the vehicles is important in VANET communication. Therefore, we propose a PoL privacy preservation mechanism that protects the privacy of vehicles. The smart contract tables are maintained by the miner nodes, and they contain vehicles’ location information. The verifiers can verify the location of the specific vehicle by querying the smart contract table without knowing the VIN number or plate number of the vehicle. The location and identity privacy of the vehicle are maintained with the proposed PoL scheme.

In the future, we will investigate the proof-of-location mechanism for cases where both single-core ECUs and multi-core ECUs are involved in the proof-of-location process among diverse vehicles in VANET.

## Figures and Tables

**Table 1:** Notations.

Notations	Description
$V_{i}$	vehicle *i*
$R_{j}$	RSU *j*
$P_{i}$	plate number of $V_{i}$
$L_{i}$	location of $V_{i}$
$S_{i}$	secret key of $V_{i}$
$t_{i}$	the time when $V_{i}$ issues its location information
*T*	difficulty target to solve the computational puzzle
$PID_{V_{i}}$	pseudo ID of vehicle $V_{i}$
$PID_{R_{j}}$	pseudo ID of RSU $R_{j}$
$VIN_{i}$	vehicle identification number of $V_{i}$
$K_{V_{i}}^{+}$	public key of $V_{i}$
$K_{V_{i}}^{-}$	private key of $V_{i}$
$K_{R_{j}}^{+}$	public key of $R_{j}$
$K_{R_{j}}^{-}$	private key of $R_{j}$
$K_{SC(R_{j})}^{+}$	public key of smart contract SC at $R_{j}$
$K_{SC(R_{j})}^{-}$	private key of smart contract SC at $R_{j}$
$K_{X_{i}}^{+}$	public key of verifier $X_{i}$
$K_{X_{i}}^{-}$	private key of verifier $X_{i}$
$Cert_{V_{i}}$	certificate of $V_{i}$
$Cert_{R_{j}}$	certificate of $R_{j}$
*H*	cryptographic hash function
$RID_{j}$	RSU ID of $R_{j}$
*g*	generator
*q*	a prime number

**Table 2:** Structure of the smart contract table to store PoL information of vehicles.

Time	Location	Z	v	Random Number for OTP
$t_{i}$	$L_{i}$	$Z_{i}$	$v_{i}$	$n_{i}$

**Table 3:** Gas cost for the proposed VANET blockchain smart contract (1 Ether = 2681.60 USD).

Function	Gas Consumed	Ether Cost (ETH)	Cost In USD
registerVehicle	92,041	0.00000114715776573	0.0031
registerRSU	92,118	0. 000000101428973069	0.00027
registerEvent	145,142	0.000000096121497696	0.00026
registerSignature	96,283	0.000000088568049906	0.00024
update_info	59,721	0.000000079384553594	0.00021
revokeVehicle	25,527	0.00000008284361536	0.00022
get	25,087	0.000000089158179441	0.00024

**Table 4:** Simulation Parameters.

Parameter	Value
Simulation area	$500\times 500\;(m^{2})$
Obstacle shadowing model	Simple obstacle shadowing
Number of vehicles	100
Number of RSUs	5
Max speed	40 km/h
Data transmission rate	6 Gbps
Wave range	40 m
Blocksize	50,000 bytes
Block time	15 s
Consensus algorithm	PoW (Proof-of-Work)
Computation power (α)	0.05
Simulation time	100 s

**Table 5:** Comparison of the proposed work with the literature.

	Sybil Attack	Privacy	DoS	Scalability	Computation Cost	Blockchain-Based	Overhead	Advantages	Limitations
**Proposed Scheme**	✓	✓	✓	✓	Low	✓	Low	scalable solution, do not impose burden to TA	single-core ECU assumption
**[7]**	✓	✗	✗	✗	Low	✗	Medium	high detection rate	burden to TA for managing the trajectories
**[8]**	✓	✗	✗	✗	Low	✓	Medium	high detection rate	burden to TA for managing the trajectories
**[9]**	✓	✓	✗	✓	Medium	✗	Medium	high detection	RSU dependent
**[11]**	✗	✓	✗	✓	Low	✓	Low	blockchain based solution	can not detect Sybil attack
**[12]**	✗	✓	✗	✓	Low	✓	Low	biometrics-based blockchain system	do not consider Sybil attack
**[13]**	✗	✓	✗	✗	High	✓	High	efficient for privacy protection	increase system overhead
**[16]**	✗	✓	✗	✓	Low	✓	Low	low overhead	can not detect Sybil attack
**[17]**	✗	✗	✗	✗	High	✓	High	decentralized approach	lack privacy and security solutions
**[19]**	✓	✓	✗	✓	Low	✓	Low	scalable solution	do not consider Sybil attack
**[20]**	✗	✗	✓	✗	High	✓	High	decentralized data collection framework	high overhead

✓ Have been studied, ✗ Have not been studied

## Data Availability

Dataset available on request from the authors.

## Abbreviations

The following abbreviations are used in this manuscript:

VANET	vehicular ad hoc network
ITS	Intelligent Transportation System
V2V	Vehicle-to-Vehicle Communication
V2I	Vehicle-to-Infrastructure Communication
V2N	Vehicle-to-Network Communication
V2P	Vehicle-to-Pedestrian Communication
V2G	Vehicle-to-Grid Communication
DSRC	Dedicated Short Range Communication
RSU	Road Side Unit
CA	Certificate Authority
OBU	On Board Unit
PoL	Proof-of-Location
PoW	Proof-of-Work
CPU	Central Processing Unit
DMV	Department of Motor Vehicles
ZKP	Zero-Knowledge Proof
TA	Trusted Authority
VIN	Vehicle Identification Number
ECU	Electronic Control Unit
